# Upfront BRAF/MEK inhibitors for treatment of high-grade glioma: A case report and review of the literature

**DOI:** 10.1093/noajnl/vdac174

**Published:** 2022-11-19

**Authors:** Gabrielle Arbour, Benjamin Ellezam, Alexander G Weil, Romain Cayrol, Magimairajan Issai Vanan, Hallie Coltin, Valérie Larouche, Craig Erker, Nada Jabado, Sébastien Perreault

**Affiliations:** Division of Child Neurology, Department of Neurosciences, CHU Sainte-Justine, Université de Montréal, Montreal, QC, Canada; Department of Pathology, CHU Sainte-Justine, Université de Montréal, Montreal, QC, Canada; Division of Neurosurgery, Department of Surgery, CHU Sainte-Justine, Université de Montréal, Montreal, QC, Canada; Division of Pathology, Department of Pathology and Cell Biology, Centre Hospitalier de l’Université de Montréal, Université de Montréal, Montréal, QC, Canada; Pediatric Neuro-Oncology, Cancer Care Manitoba and Department of Pediatrics and Child Health, University of Manitoba, Winnipeg, MB, Canada; Division of Hemato-Oncology, Department of Pediatrics, CHU Sainte-Justine, Université de Montréal, Montreal, QC, Canada; Division of Hemato-Oncology, Department of Pediatrics, Centre Hospitalier Universitaire de Québec-Université Laval, Quebec City, QC, Canada; Division of Hemato-Oncology, Department of Pediatrics, IWK Health Centre, Dalhousie University, Halifax, NS, Canada; Division of Hemato-Oncology, Department of Pediatrics, McGill University Health Center, Montreal Children’s Hospital, Montreal, QC, Canada; Division of Child Neurology, Department of Neurosciences, CHU Sainte-Justine, Université de Montréal, Montreal, QC, Canada

**Keywords:** BRAF inhibitors, *BRAF* V600E, high-grade glioma, MEK inhibitors

## Abstract

**Background:**

High-grade gliomas (HGG) with *BRAF*V600E mutation represent a unique subset of central nervous system tumors. Targeted therapies including BRAF and MEK inhibitors are now being explored as possible new treatment options.

**Methods:**

We report an 18-year-old female with a grade 3 pleomorphic xanthoastrocytoma treated upfront with dabrafenib and trametinib. We also conducted a systematic literature review of patients with HGG and *BRAF*V600E mutations treated with BRAF inhibitors.

**Results:**

Despite local recurrences resected surgically, the patient has been on dabrafenib and trametinib for more than 54 months. Thirty-two patients with HGG and *BRAF*V600E mutations treated with BRAF inhibitors were retrieved through our systematic review of the literature. Only 1 young patient with an anaplastic ganglioglioma was treated upfront with a BRAF inhibitor with a curative intent. Best response reported with radiation therapy and systemic therapy was a stable disease (SD) for 18 patients (56.3%) and progressive disease (PD) for 9 patients (28.1%). Responses to treatment regimens that included BRAF inhibitors were reported in 31 patients and included 4 complete responses (12.9%), 23 partial responses (74.2%), 2 SDs (6.5%), and 2 PDs (6.5%).

**Conclusions:**

Our patient had durable disease control with dabrafenib and trametinib. Given favorable responses reported in patients with HGG treated with BRAF inhibitors, we believe that upfront targeted therapy is a possible treatment approach that should be studied in the context of a clinical trial.

**Key Points**

- BRAF inhibitors appear to be an effective approach for the treatment of HGG with *BRAF*V600E mutation.
- BRAF inhibitors should be studied as a possible option for upfront treatment in HGG with *BRAF*V600E mutation.

**Importance of the Study**

We reported the evolution of a young patient treated upfront with a combination of BRAF/MEK inhibitors. This approach without prior chemotherapy or radiation has rarely been reported. We conducted a systematic review of the literature and report that responses are more frequent in patients treated with BRAF inhibitors when compared to standard regimens. Results from our review and our clinical experience suggest that clinical trials should investigate the possibility to use BRAF and MEK inhibitors in upfront therapy for HGG with *BRAF*V600E mutation.

*BRAF*V600E mutation is the second most frequent mutation in pediatric low-grade glioma (LGG) but the alteration has also been reported in a subset of primary and secondary high-grade glioma (HGG).^1^ The overall survival of HGG *BRAF*V600E mutation is more favorable than other subgroups including HGG H3.3 G34 R/V but despite standardized treatment strategies including surgery, radiation therapy, and chemotherapy the prognosis is poor.^2,3^ New treatment approaches are currently being investigated including BRAF inhibitors (BRAFi) with or without MEKi for LGG and HGG with *BRAF*V600E mutation. However, there are currently limited data available on the outcome of patients treated with BRAFi. Following our experience with 1 patient with HGG treated upfront with BRAFi and MEKi, we aimed to better understand the expected outcome in similar patients treated with targeted therapy by conducting a systematic review of the literature.

## Materials and Methods

We describe a case report of a young patient with a HGG treated with BRAFi and MEKi.

A systematic review of the literature in the PubMed and Embase databases was conducted for original articles on HGG including glioblastoma (GBM), anaplastic astrocytoma, anaplastic ganglioglioma, and grade 3 pleomorphic xanthoastrocytoma (PXA) with *BRAF*V600E mutations and treated with BRAFi (Supplementary Figure 1).^4^ Articles that did not meet eligibility criteria were rejected and were not included in this review, as suggested by the Cochrane guidelines. Titles and abstracts from the remaining articles were reviewed by authors (S.P. and G.A.). Relevant articles were then reviewed in detail and included. Given the nature of the study, no ethical board approval was required. The family and patient gave their consent for this case report.

## Results

### Case History

**Figure 1.**
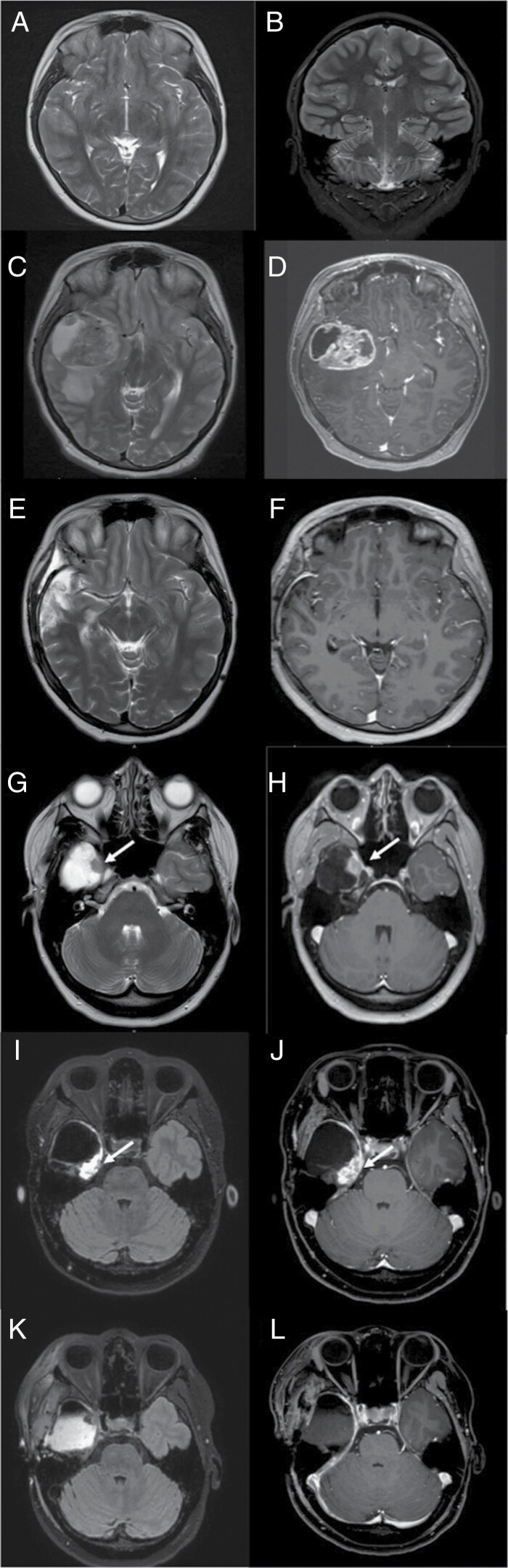
Brain magnetic resonance imaging (MRI) at the time of epilepsy diagnosis. No lesion was identified (axial T2 and coronal T2-A and B). Brain MRI 20 months later with a large right temporal lobe lesion with mass effect and vasogenic edema (axial T2 and axial T1 with gadolinium-C and D). Post-surgery brain MRI showing near total resection (axial T2 and axial T1 with gadolinium-E and F). Brain MRI demonstrating local recurrence after 32 months of treatment (arrows) (axial T2 and axial T1 with gadolinium-G and H). Brain MRI demonstrating the second local recurrence after 47 months of treatment (arrows) (axial T2 Flair and axial T1 with gadolinium-I and J). Post-surgery brain MRI showing a gross total resection (axial T2 Flair and axial T1 with gadolinium-K and L).

Our patient was diagnosed with epilepsy at 14 years of age when she presented with focal seizures characterized by “déjà vu phenomenon” followed by an alteration of consciousness. Electroencephalogram showed intermittent dysfunction in the right temporal region. Magnetic resonance imaging (MRI) at diagnosis of epilepsy was normal (Figure 1A and B). She was treated with antiseizure medications and, after failing several lines of treatment, she was well-controlled with carbamazepine and lacosamide. She had no other neurological symptoms and no developmental delay.

Sixteen months after her initial epilepsy diagnosis, she had a recurrence of focal seizures refractory to antiepileptic drug adjustments. Four months later, she presented to the emergency department with diplopia, headache, and nausea. Brain MRI showed a large 5 cm heterogenous lesion localized in the right temporal lobe with mass effect and vasogenic oedema (Figure 1C and D). Previous MRI was carefully reviewed by neuro-radiologists and there was no evidence of glioma on all available sequences including T2 Flair and diffusion. A high-grade tumor was suspected, and she underwent a near total resection (with a residue of less than 1 cm^2^) including a right-side amygdalohippocampectomy (Figure 1E and F). No complication occurred during surgery and she had no neurological deficits.

**Figure 2.**
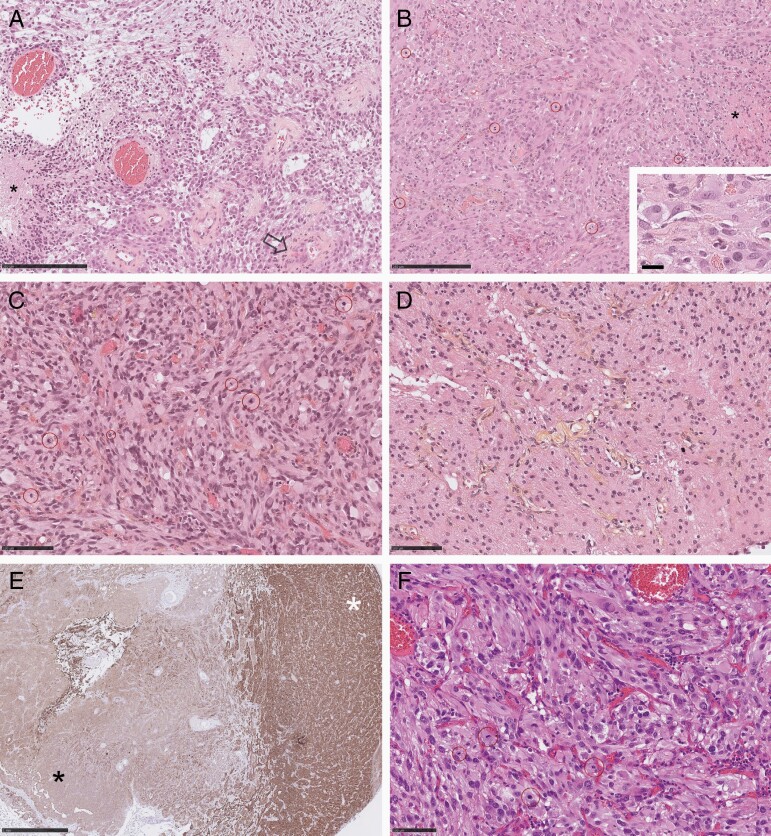
Pathology of primary tumor and recurrences. (A, B) Primary tumor showing glioblastoma-like areas with loosely arranged astrocytic tumor cells in a myxoid or microcystic background, palisading necrosis (asterisk), and microvascular proliferation (open arrow) (A, HES), and areas more typical of PXA (grade 3) with streaming fascicules of spindle cells with glassy eosinophilic cytoplasm, frequent mitoses (circled), tumor necrosis (asterisk), and xanthomatous change and eosinophilic granular bodies (inset) (B, HES). (C, E) First tumor recurrence showing PXA-like (grade 3) areas with pleomorphic astrocytic tumor cells, abundant glassy or xanthomatous cytoplasm, frequent nuclear pseudo-inclusions, and brisk mitotic activity (circled) (C, HES), and areas more typical of low-grade glioma with monomorphic tumor cells in a background of fibrillary processes, hyalinized vessel walls, and no mitoses (D, HES); immunopositivity for BRAF V600E was weaker in areas of low-grade glioma histology (black asterisk) compared to areas with features of PXA (grade 3) (white asterisk) (E). (F) Second tumor recurrence showing typical PXA (grade 3) pathology with streaming pleomorphic tumor cells, abundant glassy or xanthomatous cytoplasm, nuclear pseudo-inclusions, lymphocytic aggregates, and frequent mitoses (circled) (hematoxylin and eosin). Bar in A–B, 250 µm, inset in B, 25 µm, C, D, F, 100 µm, E, 1 mm. HES, hematoxylin-eosin-saffron.

The pathology was initially interpreted as GBM based on astrocytic morphology, necrosis, and microvascular proliferation (Figure 2A). Immunohistochemistry was positive for *BRAF*V600E mutation and comparative genomic hybridization revealed a homozygous deletion of *CDKN2A*.

Treatment options including chemoradiation therapy were presented to the family. Given the extent of resection and to avoid radiation therapy, upfront treatment with dabrafenib (150 mg by mouth twice daily) and trametinib (2 mg PO daily by mouth once daily) was initiated.

Treatment was initially well tolerated except for mild dry skin. No cardiac dysfunction was detected. After 16 months of treatment, a grade 1 retinal pigment epithelial detachment with minimal visual acuity impairment was observed. Trametinib was dose reduced by 50% to 1 mg daily.

After 32 months of treatment with targeted therapy, surveillance MRI revealed a new small nodule in the surgical cavity. A follow-up MRI 3 months later demonstrated further tumor growth and the patient underwent a second surgery (Figure 1G and H). A gross total resection was achieved. Treatment was discontinued 5 days prior to surgery and restarted 5 days after the resection. Pathology was interpreted as recurrent GBM but with prominent areas of lower-grade glioma histology (Figure 2D). *BRAF*V600E mutation was confirmed by immunohistochemistry and PCR. Given the slow progression and presence of areas with low-grade features in the recurrence, methylation profiling was requested on both the initial tumor and the recurrence. Both profiles were highly consistent with grade 3 PXA (score: 0.98). RNAseq did not identify a resistance mechanism or significant alteration such as telomerase reverse transcriptase promoter mutations. Copy number variation methylation was very similar between recurrence and diagnosis. Reexamination of primary tumor pathology revealed areas compatible with PXA including scattered eosinophilic granular bodies and xanthomatous change (Figure 2B) and grade 3 was assigned based on brisk mitotic activity (9 per 10 high power fields); Ki67 proliferation index was 12.8%. Reexamination of the recurrent tumor revealed similar findings (Figure 2C). The areas of LGG histology, however, had a proliferation index <1%, did not show features of PXA (Figure 2D), and showed weaker BRAF V600E immunopositivity (Figure 2E). The patient was restarted on dabrafenib and trametinib after surgery for disease progression at the previous doses.

The patient continued her treatment but 15 months later a surveillance MRI demonstrated a new asymptomatic local progression (Figure 1I and J). The patient underwent a gross total resection of this recurrence 2 months later (Figure 1K and L). The procedure was well tolerated without new neurological deficits. Treatment was discontinued 5 days prior to surgery and restarted 5 days after the resection. Pathology of the second recurrence revealed classic grade 3 PXA features (Figure 2F) with up to 12 mitoses per 10 high power fields, a KI67 proliferation index of 43%, and strong BRAF V600E immunopositivity. The patient was referred for focal radiation therapy with her concurrent treatment of trametinib and dabrafenib.

### Systematic Review Results

A total of 23 articles were included. Of those, 21 articles were case reports or small case series.^2,5–25^ These articles described the clinical evolution and responses of 32 patients treated with BRAFi (Tables 1–3). Median age was 22.5 years (range: 1.5–50 years). Most of these tumors were hemispheric (28/31-one unknown location) and involved the temporal lobe (18/31). Based on the pathologic report provided in the articles, 10 patients had GBM, 6 had anaplastic astrocytoma, 11 had grade 3 PXA, and 5 had anaplastic ganglioglioma. Other than *BRAF*V600E mutations most patients had limited descriptions of other molecular alterations. *CDKN2A* status was reported in ten patients and homozygous deletion was noted in 8 (80%).

**Table 1.** Patients and Tumors Characteristics, Treatments, and Follow-up

Article	Age at dx (Years), Sex	Tumor Location	Diagnosis	Initial Surgery	Radiation Therapy (Y/N)	Systemic Therapy	BRAFi/ MEKi	Response to BRAFi/MEKi	Duration of Response With BRAFi/MEKi (months)	Metastasis (Y/N)	Status at Last Follow-up
Chamberlain et al. (2013)^5^	43 M	Hemisph. Frontal	aPXA	STR	Y	First line: TMZ Second line: PVC	Vemu	PD	2	N	Dead
Robinson et al. (2014)^6^	9 M	Hemisph. Fronto-parietal	GBM epitheloid	STR	Y	First line: beva/Vorino/ Topo	Vemu	PR	7	N	Alive
Bautista et al. (2014)^8^	6 M	Thalamus	AG	STR	N	First line: Irino/Beva	Vemu	PR	UNS	Y Relapse	Dead
	1,5 F	Cerebellum	AG	STR	N	First line: BB-SFOP Second line: Topo/TMZ Third line: CP/ Doxo Fourth line: Irino/Beva	Vemu	PR	20	N	Alive
	9 F	UNS	AA	UNS	Y	First line: Topo/TMZ Second line: Irino/Beva Third line: Non-peg liposomal Doxo	Vemu	N/A	N/A	N	Dead
Usubalieva et al. (2015)^9^	35 F	Hemisph. Fronto-temporal	aPXA	NTR	Y	None	Dabra	PR	3	Y Relapse	Dead
Lee et al. (2016)^10^	43 M	Hemisph. Fronto-temporal	aPXA	UNS	UNS	First line: TMZ	Vemu	PR	3 (ongoing)	N	Alive
Meletath et al. (2016)^11^	25 M	Hemisph. Parietal	AG	STR	Y	First line: TMZ	Dabra	CR	24	N	Alive
Brown et al. (2017)^12,13^	48 F	Hemisph. UNS	aPXA	GTR	Y	First line: CCNU/VCR Second line: TMZ Third line: Carbo	Dabra Tram	PR	8 (ongoing)	N	Alive
	21 F	Hemisph. Temporal	aPXA	GTR	Y	First line: TMZ	Vemu Dabra	CR	18 (ongoing)	N	Alive
Burger et al. (2017) ^14^	24 M	Hemisph. Temporal	aPXA	GTR	Y	First line: TMZ	Dabra	PR	27	Y Relapse	Alive
	50 M	Hemisph. Temporal	AA	GTR	Y	First line: TMZ Second line: CCNU/Procarb	Dabra	PR	8 (ongoing)	Y Relapse	Alive
	25 M	Hemisph. Temporal	GBM	GTR	Y	First line: TMZ Second line: CCNU	Dabra	PR	3	Y Relapse	Alive
Johanns et al. (2018)^15^	24 M	Hemisph. Frontal	GBM epitheloid	GTR	Y	First line: TMZ Second line: Beva/TMZ	Dabra Tram	PR	4	Y Relapse	Dead
	28 F	Hemisph. Temporal	GBM epitheloid	STR	N	None	Dabra Tram	PR	11	Y Relapse	Dead
Ceccon et al. (2018) ^16^	9 M	Hemisph. Temporo- parietal	AA	UNS	Y	First line: TMZ Second line: CCNU	Dabra	SD	10	N	Dead
Marks et al. (2018)^17^	16 F	Hemisph. Temporal	AG	NTR	Y	First line: TMZ	Vemu Dabra Tram	CR	6 (ongoing)	N	Alive
Schreck et al. (2018)^7^	32 M	Hemisph. Temporal	aPXA	UNS	Y	First line: TMZ Second line: BCNU	Dabra Tram	PR	14	N	Dead
	23 F	Hemisph. Frontal	GBM epitheloid	GTR	Y	First line: TMZ	Dabra Tram	PR	UNS	N	Alive
Thomas et al. (2019)^18^	16 F	Hemisph. Frontal	aPXA	STR	Y	First line: TMZ Second line: Beva	Dabra Tram	PR	9	Y Relapse	Dead
Smith-Cohn et al. (2019)^19^	23 F	Hemisph. Temporo-parietal	aPXA	NTR	Y	First line: TMZ Second line: Beva	Dabra Tram	PR	1,5	Y Relapse	Dead
	46 M	Hemisph. Temporal	AA	GTR	Y	First line: TMZ Second line: Beva	Dabra Tram	PD	1,5	Y Relapse	UNS
Toll et al. (2019)^20^	13 M	Hemisph. Frontal	AA	GTR	Y	First line: TMZ	Dabra Tram	SD	6	Y Relapse	Dead
	12 F	Hemisph. Fronto-temporal	HGG	STR	Y	First line: Cisplat/ CP/Etopo/VCR	Dabra Tram	PR	32 (ongoing)	N	Alive
	4 F	Hypothalamus	AG	No	N	None	Dabra Tram	PR	23 (ongoing)	N	Alive
Woo et al. (2019)^21^	22 F	Hemisph. Temporal	GBM epitheloid	NTR	Y	None	Dabra Tram	PR	3	Y Diagnosis	Dead
	22 M	Hemisph. Frontal	GBM epitheloid	STR	N	None	Vemu + Cobi + Palbociclib	PR	5,5	Y Diagnosis	Dead
Pina et al. (2020)^22^	19 M	Hemisph. Parieto-temporal	aPXA	STR	Y	First line: TMZ	Dabra Tram	PR	31	N	Alive
Kushnirsky et al. (2020)^23^	44 M	Hemisph. Temporal	GBM	GTR	Y	First line: TMZ Second line: Carbo Third line: Autologous dentritic cell vaccine	Dabra Tram	CR	11 (ongoing)	N	Alive
Sen et al. (2020)^24^	37 M	Hemisph. Parietal	aPXA	UNS	Y	First line: TMZ Second line: Evero	Dabra Vemu	PR	12	N	Alive
	22 M	Hemisph. Temporal	AA	STR	Y	First line: TMZ Second line: TMZ/ Veliparib/ Carmustine/ 3rd line: retinoic acids 4th line: Evero	Vemu	PR	17	N	Dead
Venkatesh et al. (2021)^25^	19 F	Hemisph. Temporal	GBM epitheloid	GTR	Y	First line: TMZ	Dabra Tram	PR	29	Y Relapse	Dead

Abbreviations: AA, anaplastic astrocytoma; AG, anaplastic ganglioglioma; aPXA, anaplastic pleomorphic xanthoastrocytoma; BB-SFOP, carboplatin/procarbazine, cisplatin/etoposide, and vincristine/cyclophosphamide, BCNU, carmustine; BRAFi, BRAF inhibitor; Carbo, carboplatin; CCNU, lomustine; CP, cyclophosphamide; Cobi, cobimetinib; CR, complete response; Dabra, dabrafenib; Doxo, doxorubicine; Etopo, etoposide; Evero, everolimus; FU, follow-up; Hemisph., hemispheric; GBM, glioblastoma; GTR, gross total resection; Irino, irinotecan; NTR, near total resection; PD, progressive disease, PR (response to treatment), partial remission; Procarb, procarbarbazine; Pt, patient; PVC, procarbazine, vincristine, and CCNU; SD, stable disease; STR, sub-total resection; TMZ, temozolomide; Topo, topotecan; Tram, trametinib; UNS, unspecified; Vemu, vemurafenib; VCR, vincristine; vorino, vorinostat.

**Table 2.** Summarized Patients’ Characteristics

Baseline Characteristics *n* = 32 (%)	
Male	18 (56.3%)
Female	14 (43.7%)
Median age years (range: 1.5–50)	22.5
**Tumor characteristics**	
Pathological type	
GBM	10 (31.2%)
aPXA	11 (34.4%)
Anaplastic astrocytoma glioma	6 (18.8%)
Anaplastic ganglioglioma	5 (15.6%)
**Location and metastasis status**	
Hemispheric	28 (87.5%)
Temporal	16 (50%)
Frontal	12 (37.5%)
Other	3 (9.4 %)
Unknown	1 (3.1%)
Leptomeningeal dissemination	14 (43.8%)
Diagnosis	2 (14.3%)
Relapse	12 (85.7%)

Abbreviations: aPXA, anaplastic pleomorphic xanthoastrocytoma; GBM, glioblastoma.

**Table 3.** Summarized Treatment and Follow-up

Treatment	
Surgical resection	32 (100%)
Radiotherapy	28 (87.5%)
Second round radiotherapy	10 (31.3%)
Prior systemic therapies	28 (87.5%)
More than 2 lines of systemic therapies	12 (37.5%)
BRAF inhibitors	
Median time interval between systemic treatment and BRAF inhibitors months (range: 2–98)	11 months
Median time of treatment months (range 1.5–31)	9 months
BRAF inhibitors	
Combination	16 (50%)
Dabrafenib + Trametinib	15 (46.9%)
Vemurafenib + cobimetinib	1 (3.1%)
Monotherapy	16 (50%)
Dabrafenib	7 (21.9%)
Vemurafenib	7 (21.9%)
Vemurafenib followed by dabrafenib	2 (6.3%)
Reported best response to BRAF inhibitors	
CR	4 (12.5%)
PR	23 (71.9%)
SD	2 (6.3%
PD	2 (6.3%)
Final outcome reported after initiation of BRAF inhibitors	
Progressive disease	29 (90.6%)
Death	16 (50%)
Median Survival OS range months	24 months (2–112 mths)

Abbreviations: CR, complete response; GTR, gross total resection; NTR, near total resection; PD, progressive disease; PR (response to treatment), partial remission; SD, stable disease.

Progressive disease (PD) was reported in 29 patients (90.6%) and death was reported in 16 patients (50 %) with a median overall survival of 24 months after diagnosis (range 2–112 months). All patients died of their disease except for 1 patient who suffered from an extensive intracerebral hemorrhage related to vemurafenib according to the authors.^8^ Fourteen (43.8%) had leptomeningeal dissemination at 1 point during their follow-up including 2 patients at diagnosis.

In terms of treatment, 26 (83.8%, 26/31) had focal radiation therapy following resection and 10 patients (31.3%) received a second round of focal radiation therapy. Five patients did not receive radiation therapy: 3 patients due to poor neurological status and 2 patients due to young age (1.5 and 4 years old). Most patients also received prior systemic treatment before BRAFi (81.3%, 26/32); 12 patients (37.5%) had received more than 2 lines of systemic therapy (range 1–4). Only one young patient with an anaplastic ganglioglioma was treated upfront with a BRAFi with a curative intent.^20^ Best response achieved on systemic therapy before BRAFi (based on MRI/ and clinical assessment) was a stable disease (SD) for 18 patients (56.3%) and PD for 9 patients (28.1%).

Median time between diagnosis and the start of treatment with BRAFi was 11 months (range 2–98 months). The treatment regimens varied: 15 (46.9%) patients were treated with combination of dabrafenib plus trametinib; 7 (23.3%) patients received dabrafenib monotherapy; 7 (23.3%) patients were treated with vemurafenib monotherapy; 2 (6.7%) patients received vemurafenib followed by dabrafenib; 1 (3.3%) patient received combination of vemurafenib plus cobimetinib. Median treatment time with BRAFi was 9 months (range 1.5–32 months) and 7 (23.3%) patients were still receiving targeted therapy at the time of reporting. Response was reported in 31 patients and included 4 complete responses (CRs; 12.9%), 23 partial responses (PRs; 74.2%), 2 SDs (6.5%), and 2 PDs (6.5%).

Two clinical trials involving patients with HGG and *BRAF*V600E mutations treated with BRAFi in monotherapy or the combination of BRAFi and MEKi were also reviewed.^26,27^ Kaley et al. reported 24 adults with CNS tumors and *BRAF*V600E mutations treated with vemurafenib including 15 patients with HGG including 6 patients with GBM, 5 with anaplastic astrocytoma, 3 anaplastic gangliogliomas, and 1 HGG not otherwise specified. All patients with high-grade tumors had received prior treatment including radiation therapy (15/15) and most had received chemotherapy (12/15). Using the response evaluation criteria in solid tumors 1.1 criteria, 2 patients had a PR (13.3%, 2/15). They reported a median overall survival for GBM and anaplastic astrocytoma of 11.9 months.^26^ In a recent study by Wen et al., adult patients with *BRAF*V600E mutation were treated with combination of dabrafenib and trametinib.^27^ The study included 45 HGG (31 GBM). All patients received prior therapy including radiation therapy followed by chemotherapy or concurrent chemoradiotherapy. They observed an overall response rate (ORR) of 33% with 3 CR and 12 PR. SD was seen in 22% and PD in 42% of patients. Median duration of treatment was 14.9 months for HGG. Median overall survival was 13.7 months for GBM and 45.2 months for other HGG.^27^

## Discussion

We report a case of a teenager with a grade 3 PXA who initially presented with focal seizures. Her first MRI was normal, and the lesion was identified 16 months later when seizures recurred with symptoms of intracranial hypertension. The patient was treated upfront with a combination of dabrafenib and trametinib. We conducted a systematic review of the literature and summarized current data on treatment with BRAFi of HGG with *BRAF*V600E mutation.

In children, only 1%–3% of patients with epilepsy have an underlying tumor.^28^ The initial MRI as part of the epilepsy workup usually reveals the tumor but in rare cases, the lesion is not detected. In an adult series of patients with HGG, 4.7% had normal imaging at presentation (9/193).^29^ MRIs are usually repeated in patients with refractory epilepsy, especially if they were well controlled in the past or if they presented with new neurological symptoms to rule out an ongoing process not initially recognized.

Given the extent of resection, tumor location, and family preference to avoid radiation therapy, we decided to treat this patient upfront with dabrafenib and trametinib without radiation. This approach has rarely been reported. In our systematic review, only 4 patients received upfront BRAFi including 3 patients with poor neurological status precluding the use of radiation and chemotherapy. Only 1 young patient with an anaplastic ganglioglioma was treated upfront with a BRAFi with a curative intent. The patient has been stable for more than 23 months according to the authors.^20^

Responses to BRAFi in patients with HGG vary in the literature. In our literature review, 27 patients (87.1%) presented a significant decrease in their tumors (clinically assessed as PR). This high response rate needs to account for a possible publication bias toward selection of case reports with favorable responses to BRAFi. However, other studies have revealed a high rate of response in HGG with *BRAF*V600E mutation.

Nobre et al. reported a large cohort of patients with gliomas and *BRAF*V600E mutations treated with BRAFi.^2^ A total of 11 children with HGG were reported (6 GBM, 2 anaplastic gangliogliomas, 2 grade 3 PXA, and 1 anaplastic astrocytoma). Detailed treatment and disease course were not provided but all patients received prior radiation therapy and 9 received between 1 and 3 lines of systemic therapy. Four patients (36%) responded (1 CR and 3 PRs) and all patients except 1 progressed within 18 months. Median time to progression was 10 months.

Recently, Rosenberg et al. reported an original series of 19 pediatric patients with HGG and *BRAF*V600E mutations. Sixteen (84.2%) received upfront radiation therapy followed by BRAFi+/−MEKi and 3 patients underwent biopsy with upfront treatment with BRAFi+/−MEKi. They reported an ORR of 64.3% when including CR and PR. Only 1 patient had PD as the best response. They showed a favorable 18-month progression free survival (PFS) of 83% compared to 42% in the BRAF-mutant historical cohort. They reported a 3-year overall survival (OS) of 82% compared to 44% in the BRAF-mutant historical cohort.^30^

Andrews et al also conducted a large systematic review of glioma with *BRAF*V600E mutation. They reported an ORR of 56% in patients with pediatric HGG and 38.2% in patients with adult HGG treated with BRAFi+/−MEKi.^31^

Recently, Hargrave et al. presented the results of the recently completed Novartis trial for pediatric HGG with *BRAF*V600E mutations (NCT02684058). After completing at least 1 line of treatment (radiation and/or chemotherapy) patients received a combination of dabrafenib and trametinib. The ORR was 56.1% for the entire cohort and 66.7% for PXA grade 3 (4/6 patients). The 12 months PFS was 44.1% and the OS was 32.8%.^32^

In recurrent HGG, response rates with chemotherapy have rarely exceeded 5%, with an overall survival of 5–9 months and progression-free survival of less than 3 months.^33^ Therefore, the use of BRAFi might be more effective with at least transient improvement in tumor control, neurological function, and quality of life. Given the small number of patients in our literature review, we did not observe significant differences in response rates between pediatric patients, adolescents/young adults, and adults. It will be interesting to see if upcoming studies report different outcomes based on age.

It is possible that prior treatments negatively impact the outcome of these patients. Radiation and chemotherapy could induce new mutations facilitating resistance to targeted therapy. Data are currently very limited to support this hypothesis but given the poor outcomes of patients with HGG, new treatment approaches (including upfront targeted therapy) and a modification of sequences of treatment should be considered. Treatment with BRAFi could be initiated at diagnosis and radiation therapy with chemotherapy could be reserved for progression. In some cases, targeted therapy could be continued during radiation and chemotherapy in select patients to avoid the rebound phenomenon.^34^ This should be investigated in the setting of a clinical trial. However, given the rarity of HGG with this alteration, it may take several years and the involvement of a large consortium to answer this question. Currently, the Children’s Oncology Group is conducting a study using dabrafenib and trametinib for HGG following local radiation therapy (NCT03919071).

In 16 patients (50%) a combination of BRAFi and MEKi were used. The benefit of this combination for HGG have not been determined yet but studies in adults with melanoma have reported a reduction in both death and progression with similar toxicity.^35,36^ The combination of dabrafenib and trametinib was used in the Novartis trial for HGG (NCT02684058).^32^ Despite limited data, we used this approach to optimize tumor control.

In our literature review, leptomeningeal dissemination was frequently reported in patients with HGG and *BRAF*V600E mutation (14/30, 46.7%). This is higher than what is generally, reported in GBM where approximatively 25% of patients have metastases within the central nervous system at 1 point during their follow-up.^37^ It will be important to investigate whether HGG with *BRAF*V600E mutations are more likely to have leptomeningeal dissemination in future large studies.

Another limitation of this systematic review of the literature is the accuracy and classification of diagnosis. Diagnoses were based on descriptions and not in accordance with the latest WHO classification of CNS tumors.^38^ The term anaplastic is no longer used for diffuse astrocytoma and it was not possible to know if some of these tumors would be classified differently. Nevertheless, we included case reports of patients with features suggestive of higher-grade lesions that have been historically treated aggressively with resection, radiation, and chemotherapy.

## Conclusion

We report a case of grade 3 PXA treated upfront with dabrafenib and trametinib. Despite local recurrences, our patient had a favorable and durable disease control. We reviewed the current literature, and this treatment approach has rarely been reported. Given the favorable response seen in patients with HGG treated with the combination of BRAFi and MEKi, we suggest that upfront targeted therapy treatment is feasible and should be studied in the context of a clinical trial.

## Supplementary Material

vdac174_suppl_Supplementary_Figure_S1
