# Supplementary material for: Upfront BRAF/MEK inhibitors for treatment of high-grade glioma: A case report and review of the literature
Source: Neurooncol Adv. 2022 Nov 19;4(1):vdac174. doi: 10.1093/noajnl/vdac174 (PMC9772816; doi:10.1093/noajnl/vdac174)
Supplement: vdac174_suppl_Supplementary_Figure_S1 [file vdac174_suppl_supplementary_figure_s1.docx]

**Supplementary Figure 1**

**Identification of studies via databases and registries**

Articles identified from*:

Databases (n = 2)

Pubmed-Embase

**Identification**

Articles screened

(n = 75)

**Screening**

Articles excluded:

Reason 1 (n =20) (Low grade tumors)

Reason 2 (n = 2) (Adverse effects)

Reason 3 (n = 12) (Extra-cerebral tumors)

Reason 4 (n= 17) (Articles on theory, no case reported)

Reason 5 (n=1) (Article with cases from literature review)

Articles assessed for eligibility

(n = 75)

**Included**

Articles included in review

(n = 23)

An initial search was performed on the July 16^th^, 2021 with the support of the librarian at CHU Sainte-Justine Medical Library. An updated search was done on November 15^th^ 2021

Braf AND mutant OR v600 AND (dabrafenib OR trametinib OR vemurafenib) AND ("high grade" OR astrocytoma OR Glioblastoma OR “anaplastic ganglioglioma” OR “anaplastic xanthoastrocytoma”)

Langage included: English, French, Spanish
